# Efficient SARS-CoV-2 detection in unextracted oro-nasopharyngeal specimens by rRT-PCR with the Seegene Allplex™ 2019-nCoV assay

**DOI:** 10.1186/s12985-020-01468-x

**Published:** 2020-12-18

**Authors:** Wesley Freppel, Natacha Merindol, Fabien Rallu, Marco Bergevin

**Affiliations:** 1 Institut national de la recherche scientifique, Centre Armand-Frappier Santé Biotechnologie, Laval, QC H7V 1B7 Canada; 2 Département de chimie, biochimie et physique, Université du Québec à Trois-Rivières, Trois-Rivières, QC Canada; 3 Centre Intégré Universitaire de santé et services sociaux de la Mauricie et Centre du Québec, Trois-Rivières, QC Canada; 4 Microbiology Department, Sainte-Justine Mother and Child University Hospital, Montréal, QC Canada; 5 Département de biologie médicale Hôpital Cité-de-la-Santé, Laval, QC H7M 3L9 Canada

**Keywords:** SARS-CoV-2, rRT-PCR, RNA, COVID-19, Detection

## Abstract

**Background:**

The fight against the COVID-19 pandemic has created an urgent need to rapidly detect infected people. The challenge for clinical laboratories has been finding a high throughput, cost-efficient, and accurate testing method in the context of extraction reagents shortage on a global scale. To answer this need, we studied SARS-CoV-2 detection in oro-nasopharyngeal (ONP) swabs stored in Universal Transport Media (UTM) or in RNase-free water by rRT-PCR with Seegene Allplex™ 2019-nCoV assay without RNA extraction.

**Results:**

Optimal results were obtained when swabs stored in UTM were diluted 1/5 and 1/2 in RNase-free water. Thermal lysis before rRT-PCR testing slightly improved detection rate. In addition, proteinase K (PK) treatment allowed for a significant reduction of invalid results and increased sensitivity for detection of low viral load specimens. In a panel of positive samples with all 3 viral genes amplified and N gene Cycle threshold values (C_t_ values) from 15 to 40, our detection rate was 98.9% with PK and 94.4% without. In a challenging panel of low positive samples with only the N gene being detectable at C_t_ values > 30, detection rate was increased from 53.3 to 76.7% with the addition of PK, and invalid rate fell off from 18.3 to 0%. Furthermore, we demonstrated that our method reliably detects specimens with C_t_ values up to 35, whereas false negative samples become frequent above this range. Finally, we show that swabs should be stored at − 70 °C rather than 4 °C when testing cannot be performed within 72 h of collection.

**Conclusion:**

We successfully optimized the unextracted rRT-PCR process using the Seegene Allplex™ 2019-nCoV assay to detect SARS-CoV-2 RNAs in nasopharyngeal swabs. This improved method offers cost savings and turnaround time advantages compared to automated extraction, with high efficiency of detection that could play an important role in the surveillance of Covid-19.

## Introduction

In December 2019, the world witnessed an unknown coronavirus emerging in Wuhan, China, first called 2019 novel coronavirus (2019-nCoV), and then severe acute respiratory syndrome-coronavirus 2 (SARS-CoV-2) [1]. SARS-CoV-2 is a positive-sense single-stranded RNA virus belonging to the *Sarbecovirus* subgenus. Based on the limited scale of the outbreaks of its predecessors SARS-CoV-1 in 2003 and MERS-CoV in 2012, SARS-CoV-2’s spread around the world was unfortunately underestimated. In November 2020, 52.5 million of people have been infected. The rapid increase of daily cases worldwide has made imperative the development of fast and accurate diagnostic tools of SARS-CoV-2 in order to isolate infected people quickly to reduce transmission. Accurate and fast detection also allows for a better understanding of viral transmission rate. The usual method used for *Sarbecovirus* RNA detection involved reverse transcription polymerase chain reaction (rRT-PCR) on viral genes [2]. As a coronavirus, SARS-CoV-2 contains 4 structural proteins (S spike, M membrane, E envelope and N nucleocapsid) and several non-structural proteins (such as the RNA-dependent RNA polymerase (RdRP) responsible for the synthesis of viral RNAs), all produced from cleavage of ORF1a and ORF1b [3].

In the context of the current pandemic, our hospital laboratory receives more than a thousand samples in UTM or in RNase-free water each day for testing. Procedures that include RNA extraction are not allowing this kind of throughput with the equipment at hand. Moreover, RNA extraction kits are in limited supply due to high demands. Performing direct rRT-PCR, without any RNA extraction step, offers a quick and cost-efficient solution. However, our concern was that specimen itself or transport media such as UTM would interfere with rRT-PCR efficiency. UTM is a stable viral transport medium allowing collection and transport of viruses and bacteria that contains protective proteins and antimicrobials, which could interfere with rRT-PCR enzymes.

The first objective of this study was to address the feasibility of direct rRT-PCR and to explore the requirements of specimen dilution and PK treatment to reduce rRT-PCR interference. PK is a well-known enzyme that has several activities such as protein denaturation and nuclease inhibition and has already been shown to improve SARS-CoV-2 detection [4–6].

In this study, we have optimized a method to detect SARS-CoV-2 from nasopharyngeal swabs in UTM or in RNase-free water by amplifying E, N and RdRP genes using rRT-PCR without requiring a RNA extraction step. We demonstrate that PK addition improves detection sensitivity and reduces rates of invalid results. Finally, we also show that non- extracted samples can be analysed within 3 days if stored at 4 °C without altering rRT-PCR sensitivity.

## Material and methods

### Reagents and sample

ONP swabs were collected either in 2 ml of UTM® (COPAN) or RNase-free water and stored at 4 °C before analysis. No patients were specifically recruited, and only samples already known as positive or negative were used in this study. Poly A (Millipore Sigma ref P9403), β-mercaptoethanol **(**Gibco™ ref 31350010), proteinase K solution 20 mg/ml (ThermoFischer Scientific ref 25530049).

### Sample sets and experiments

All sample sets are summarised in Table 1.

1. 30 known positive ONP specimens collected with flocked swabs in 3 ml UTM with all three viral genes detected. These were retested by rRT-PCR after RNA extraction as standard reference and tested by direct rRT-PCR with and without thermal lysis at the following concentrations: undiluted, 1/2, 1/5, 1/10 diluted in RNase-free water. The results obtained by direct rRT-PCR were compared to those obtained after extraction.
2. 30 known positive ONP specimens collected with flocked swabs in 2 ml RNase-free water with all three viral genes detected. These were retested by rRT-PCR after RNA extraction as standard reference and tested by direct rRT-PCR with or without thermal lysis at the same dilutions mentioned above and compared to the standard reference.
3. 90 known positive ONP specimens collected with flocked swabs in 2 ml RNase-free water with all three viral genes detected with a wider range of C_t_ values than the previous sample sets (N gene C_t_ values from 15 to 40).
4. 60 known positive ONP specimens collected with flocked swabs in 2 ml RNase-free water with only N gene detected with low (C_t_ values 30–35) to very low viral loads (C_t_ values ≥ 36).
5. 60 known negative ONP specimens collected with flocked swabs in 2 ml RNase-free water.

**Table 1:** Sample sets characteristics

Sample set	1	2	3	4	5
n specimens	30	30	90	60	60
Previous rRT-PCR	+	+	+ (3 viral genes)	+ (N gene only)	-
C_t_ range N gene	random	random	Wide 15–40	Low 30–35 Very low 36–40	not detected
Medium	UTM	RF water	RF water	RF water	RF water
Storage	− 70 °C	− 70 °C	− 70 °C	− 70 °C	− 70 °C
Thermal lysis	Y vs. N	Y vs. N	Y	Y	Y
RNA extraction	Y vs. N	Y vs. N	N	N	N
Direct rRT-PCR	Y	Y	Y	Y	Y
Dilution in RF water	1/1 vs. 1/2 vs. 1/5 vs. 1/10	1/1 vs. 1/2 vs. 1/5 vs. 1/10	1/2	1/2	1/2
Proteinase K	N	N	Y vs. N	Y vs. N	Y vs. N

*RF* RNase-free, *Y* yes, *N* no

The specimens in sample sets 3, 4 and 5 (kept at − 70 °C) were retested by direct PCR, at the optimal dilution of 1/2, with thermal lysis, either with or without PK treatment prior to thermal lysis. Experiments on sample sets 3, 4 and 5 were designed to further evaluate the performance of our optimized direct rRT-PCR on a larger group of ONP specimens collected in water and to evaluate the impact of PK pre-treatment on sensitivity and reduction of invalid results.

### RNA extraction

OMEGA BIO-TEK E.Z.N.A.® Total RNA Kit I (R6834) protocol for manual extraction of viral RNAs was used. Briefly, 150 μl of nasopharyngeal swab from UTM or RNase-free water were added to 500 μl of TRK lysis buffer supplemented with carrier RNA (10 μg/ml) and β-mercaptoethanol (1 mM) into a 1.5 mL microcentrifuge tube. Tubes were vortexed for 30 s and kept at room temperature for 5–10 min. 350 μl of 100% ethanol were then added and tubes were vortexed for 30 s. Samples were transferred (including any precipitate) to a HiBind® RNA Mini Column. Columns were centrifuged at maximum speed (≥ 13,000 g) for 15 s. Columns were then washed once with 500 μl of RNA Wash Buffer I and twice with 500 μl of RNA Wash Buffer II at 10,000*g* for 1 min. Columns were centrifuged one last time at maximum speed (≥ 13,000*g*) for 2 min to remove residues. Columns were transferred into clean nuclease-free 1.5 ml tubes. 40–70 μl Nuclease-free Water was added directly on membranes into columns for 1 min and then columns were centrifuged at maximum speed (≥ 13,000*g*) for 2 min. Eluted RNAs were stored at -70 °C.

### Reverse transcriptase-polymerase chain reaction

Non-extracted samples were diluted at 1/1, 1/2, 1/5 and 1/10 in RNase-free water in a 96-well PCR plates. RNA extracted samples were used undiluted. Plates were then either stored at 4 °C while preparing master mix or heated at 90 °C for 3 min to perform thermal lysis and cooled down at 4 °C. Allplex™ 2019-nCoV assay from Seegene Inc. were used according to the manufacturer protocol to perform rRT-PCR. Briefly, for one reaction: 5 μl of 2019-nCoV MOM (MuDT* Oligo Mix (MOM):—Amplification and detection reagent *MuDT is the brand name of Seegene’s oligo mixture), 5 μl of buffer 5 ×, 5 μl of RNase-free water, 1 μl of internal control (IC) and 2 μl of enzymes. 18 μl of master mix were distributed in each well and added with either 8 μl of sample, 8 μl of positive control or 8 μl of RNase-free water for negative control (final volume of 26 μl). Plates were then spun down at 2500 rpm for 5 s and analyzed on a CFX96 Touch Real-Time PCR from BioRad. Reverse Transcription reaction 1 cycle: 50 °C/20 min – 95 °C/15 min. PCR reaction 45 cycles: 94 °C/15 s – 58 °C/30 sec. Gene amplifications were analyzed by FAM (E gene), HEX (IC), Cal Red 610 (RdRP) and Quasar 670 (N gene) fluorophores. Results were compiled and analyzed using 2019-nCoV viewer from Seegene Inc. according to the manufacturer’s instructions [7] (Table 2).

**Table 2:** Allplex™ 2019-nCoV assay interpretation

	HEX (IC)	FAM (E gene)	Cal Red 610 (RdRP gene)	Quasar 670 (N gene)	Interpretation
Case 1	±	+	+	+	2019-nCoV detected
Case 2	±	+	−	+	2019-nCoV detected
Case 3	±	+	+	−	2019-nCoV detected
Case 4	±	−	+	+	2019-nCoV detected
Case 5	±	−	−	+	2019-nCoV detected
Case 6	±	−	+	−	2019-nCoV detected
Case 7	±	+	−	−	Presumptive positive
Case 8	+	−	−	−	Negative
Case 9	−	−	−	−	Invalid

Ct ≤ 40 = detected (+); Ct > 40 = non detected (−); Ct IC > 40 = invalid (−)

### PK treatment

PK was directly added in RNase-free water prior to dilution 1/2 at a concentration of 200 µg/ml (final concentration after dilution 1/2 is 100 µg/ml). Microwell plates were then heated in a thermal cycler at 50 °C for 15 min to perform enzyme activities and then heated at 90 °C for 3 min for inactivation.

### Thermal lysis

A heat shock treatment in which the specimen is brought to 90 °C for 3 min followed by a rapid cooling step at 4 °C prior to the rRT-PCR process.

### Experimental design and statistical analyses

All sample sets were divided in 3 groups and analysed in an independent manner. Statistical significance was evaluated by Multiple *t*-test using GraphPad Prism 8.0 software. *p* value < 0.05 was considered significant: ****: *p* < 0.0001; ***: *p* < 0.001; **: *p* < 0.01; * *p* < 0.05. ΔC_t_ means N gene were referenced to compare 2 different groups by calculating the difference between the N gene C_t_ values means.

## Results

### Dilution of UTM samples improves rRT-PCR sensitivity

**Fig. 1:**
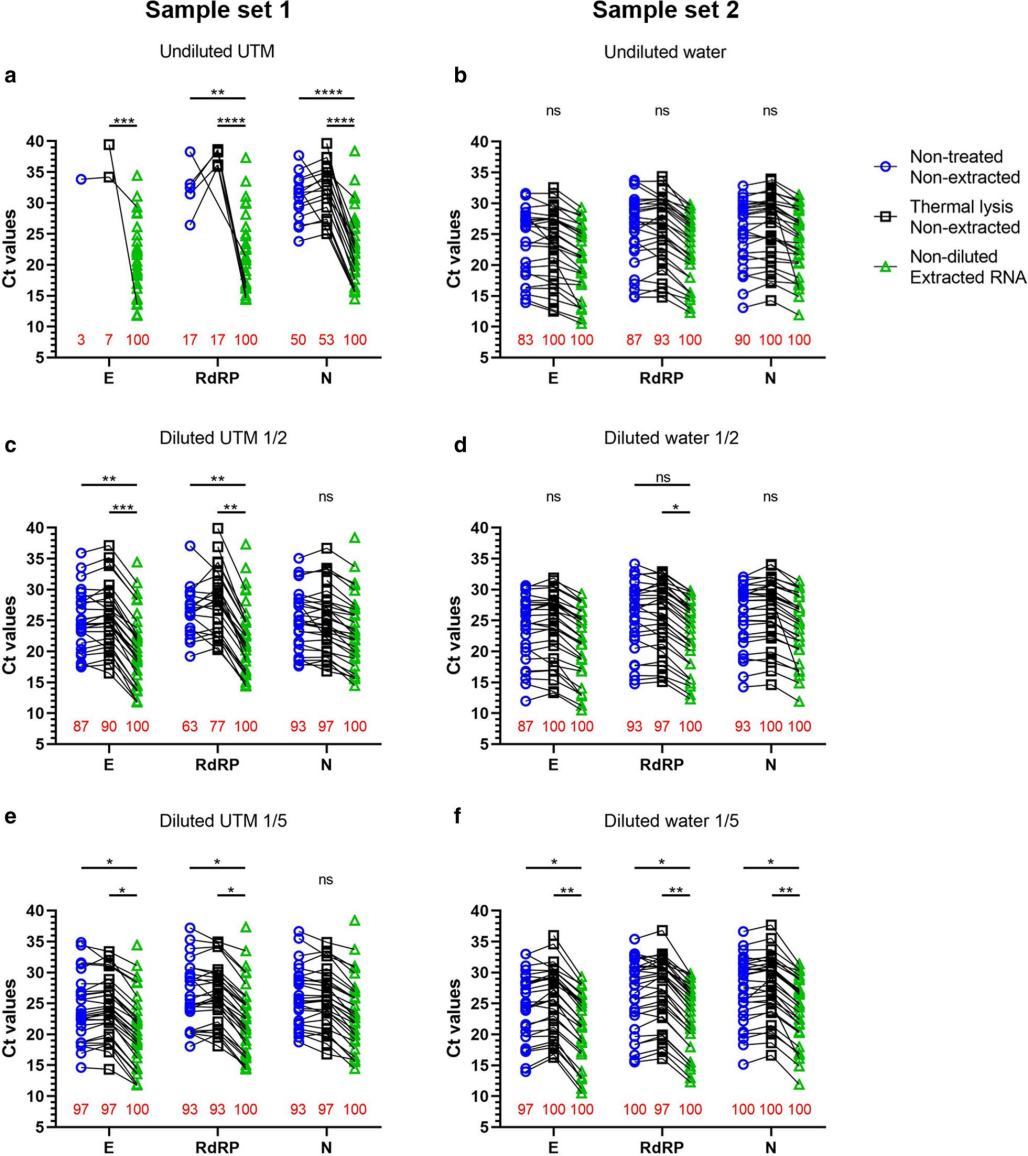
Dilutions and thermal lysis mimic RNA extraction values. 60 known positive samples in UTM (n = 30) and in RNase-free water (n = 30) were divided in 3 equal groups and analysed in 3 independent experiments by rRT-PCR on E, RdRP and N viral genes. Undiluted extracted samples were used as reference values. Red numbers indicate the percentage of detected samples. **a**, **b** samples were directly used without any dilution, with or without thermal lysis treatment. **c**–**f** Samples were diluted in 1/2 and 1/5; with or without thermal lysis treatment

With sample set 1, we compared results obtained from extracted rRT-PCR with direct rRT-PCR at various dilutions, with or without thermal lysis treatment (Fig. 1a, c, e). When using the standard reference, the range of C_t_ values was 14 to 39 with a mean value of 23.33. Our data show that direct use of undiluted UTM without RNA extraction interferes with the rRT-PCR, since only 50% (15/30) were detected positive (Fig. 1a). Sensitivity was relatively well restored when samples were diluted in RNase-free water (Fig. 1c, e). Specimen dilutions 1/2 and 1/5 showed a positive concordance of 93%, but the 1/5 dilution increased the detection rate for E and RdRP genes (+ 10% and + 30% respectively). Further diluting samples to 1/10 (data not shown) lead to a loss in detection (E: − 7%; RdRP: − 16%). These data suggest that direct rRT-PCR from UTM samples should be performed at a dilution of 1/5. At this dilution, only 2/30 patient specimens were missed, with N gene C_t_ values of 30.76 and 38.42 obtained with our reference standard. Thermal lysis before rRT-PCR testing slightly improved our detection rate for the N gene at the 1/5 dilution (+ 4%) by recovering only the missed specimen with the N gene C_t_ value of 30.76. The average N gene ΔC_t_ for our 1/5 dilution with thermal lysis as compared to our standard reference was 2.7.

### RNase-free water as media improves unextracted rRT-PCR sensitivity

Experiments with sample set 2 were performed in an identical manner to sample set 1 except that the ONP samples were collected in RNase-free water (Fig. 1b, d, f). When using the reference standard, the range of C_t_ values was 11–32 with a mean value of 24.06. We reached 90% detection rate with undiluted samples in RNase-free water. We evaluated if dilutions could improve the direct rRT-PCR efficiency as observed for UTM samples. Once again, both dilutions 1/2 and 1/5 showed greater positive concordance (93% and 100% respectively). However, the 1/2 dilution demonstrated C_t_ values closer to the ones observed following RNA extraction (ΔC_t_ mean N gene: 1/2 = 1.85; 1/5 = 3.24). Detection using 1/10 diluted specimen (data not shown) was also sensitive (97%) but with C_t_ values higher than dilution 1/2 (ΔC_t_ mean N gene: 3.35). This data suggests that RNase-free water as collection medium allows for direct rRT-PCR with results approaching those of RNA extracts, and that specimen dilution further improved the sensitivity of this approach. Finally, thermal lysis improved our detection rates to 100% in undiluted and diluted specimens without modifying the C_t_ values and also improved detection rates for the E and RdRP genes.

### Proteinase K allowed 100% valid results and improves detection of low viral load samples

**Fig. 2:**
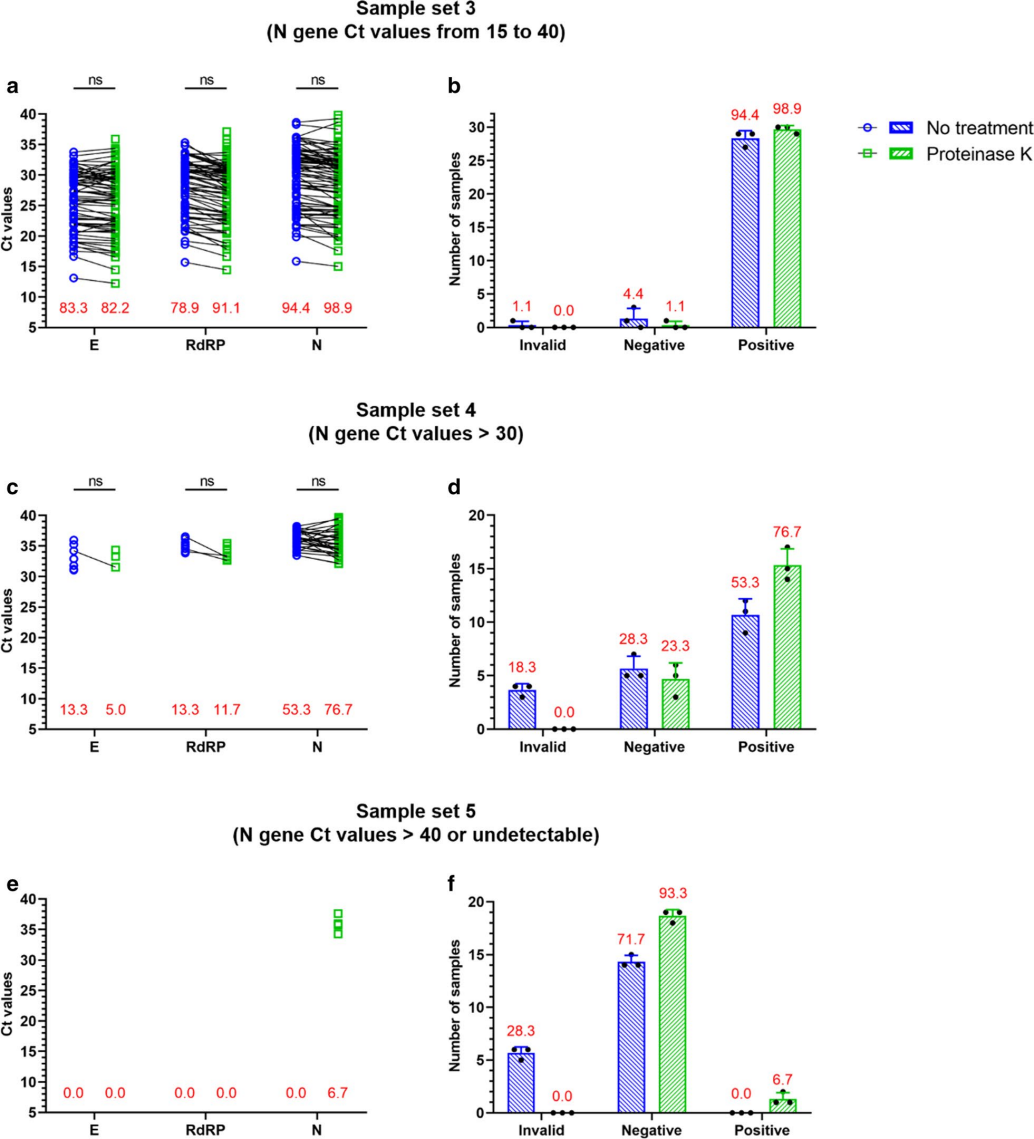
Proteinase K improves efficiency and accuracy of detection. 90 known positive RNase-free water samples for three viral genes (**a**, **b**), 60 known RNase-free water positive samples for N gene only (**c**, **d**), and 60 known RNase-free water negative samples (**e**, **f**) were divided in 3 equal groups and analysed in 3 independent experiments by rRT-PCR on E, RdRP and N viral genes with dilution 1/2 and thermal lysis treatment and with or without a PK treatment at 100 µg/ml**.** Red numbers indicate the percentage of detected samples. **a**, **c**, **e** Comparison of Ct values of viral genes. **b**, **d**, **f** Distribution of invalid, negative, or positive results. Dots represent the mean of three random groups of 30 samples each (Set 3) or 20 samples each (set 4 and 5)

Sample set 3 consisted of 90 known positives, with all three viral genes detected, and with N gene C_t_ values ranging from 15 to 40 with a mean of 29.74 (Fig. 2a, b). The detection rate achieved 98.9% (89/90) following the addition of PK compared to 94.4% (85/90) without. Three negative specimens in absence of PK (3.3%) were detected positive with C_t_ values > 37 following PK addition. The one negative specimen in both conditions (1.1%) was retested with manual extraction and was still found negative. One invalid result was obtained in the arm without PK (1.1%) and none with PK.

Sample set 4 consisted of 60 known positive samples detected solely with the N gene and with C_t_ values > 30 (Fig. 2c, d). In this challenging set, the detection rate in presence of PK was 76.7% (46/60) vs. 53.3% (32/60) without, and this difference was mostly due to PCR inhibition, as attested by the 18.3% invalid rate in the latter group. The range of C_t_ values previously obtained for the 14 negative specimens with PK treatment was 36–40. We then further investigated them following manual RNA extraction and 9/14 were detected positive with N gene C_t_ values > 35 whereas 5 samples were still detected as negative. Although PK treatment did not improve C_t_ values (ΔC_t_ mean N gene: 0.46; 0.02; Fig. 2a, c respectively), it improved detection of specimens of low viral load. In fact, PK treatment revealed 4/90 and 14/60 positives in sample set 3 and 4 respectively with only the N gene at C_t_ values mean = 37.18 ± 2.15.

Sample set 5 consisted of previously tested negative samples and was analysed mainly for the impact of PK treatment on the production of invalid results (Fig. 2e, f). Without PK treatment, 28.3% (17/60) results came out invalid following direct rRT-PCR, whereas there were no invalids following PK addition. Interestingly, we detected 4 positives in the arm with PK that had previously been negative. The C_t_ range for these positives was 34 to 38. This suggested a 6.7% false negative rate in our panel set 5 that could be detected following addition of PK by direct rRT-PCR (Fig. 2e, f).

**Fig. 3:**
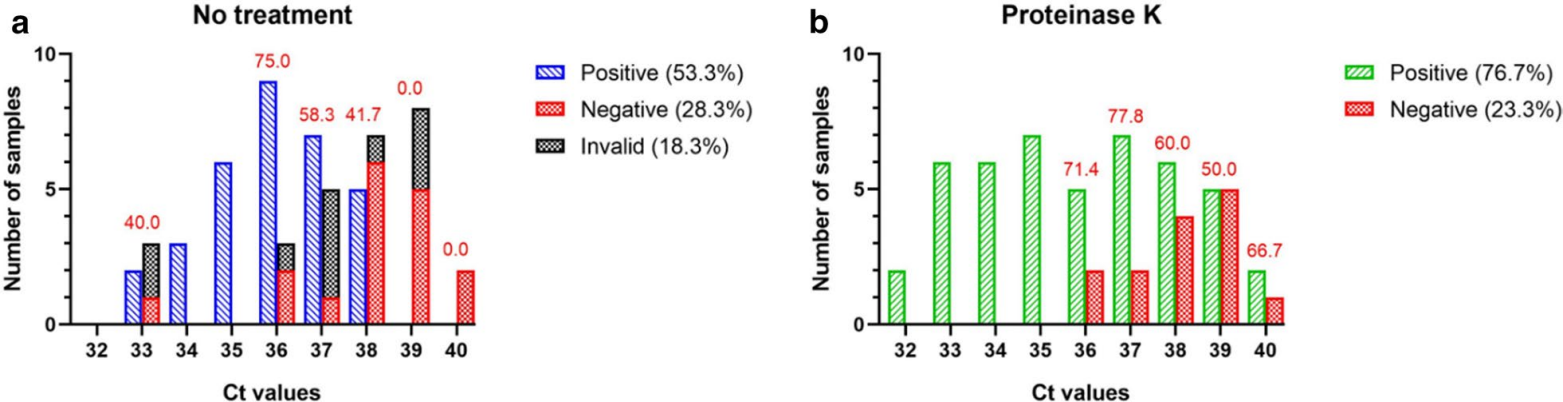
Detection efficiency over Ct values. Results from direct rRT-PCR of sample set 4 were sorted by N gene C_t_ values and with or without a PK treatment. **a** C_t_ values of invalid results were taken after detection with PK. C_t_ values of negative results were taken from previous positives analyses or after detection with PK. **b** C_t_ values of negative results were taken from previous positive analyses. Red numbers indicate the percentage of detection for each C_t_ value

In order to evaluate the threshold of the rRT-PCR, we compared the frequency of invalid, negative, and positive results from panel set 4 sorted by C_t_ values with or without PK treatment (Fig. 3). Interestingly, detection started to wane at C_t_ values of 36 in the PK arm, suggesting that our method of PK addition reaches a limiting threshold at a C_t_ value of 35.

We conclude that PK treatment increases sensitivity and reduces the rate of invalid results for direct rRT-PCR on ONP samples collected in RNase free water.

### Analytical sensitivity

A previous study was performed to determine the limit of detection (LoD) of the Allplex™ 2019-nCoV Assay using a reference RNA material [7]. The LoD was evaluated at 4167 copies/ml (viral load per millilitre) for the gene N using a CFX96™. In order to evaluate the LoD of the Allplex™ 2019-nCoV Assay on our optimized method, we analyzed in triplicate direct rRT-PCR with dilution 1/2 and PK treatment a panel of 7 samples from the Laboratoire de Santé Publique du Québec. These 7 samples consisted of SARS-CoV-2 particles resuspended in RNase-free water at different concentrations (23; 45; 90; 180; 360; 720 and 3,600 copies/ml). Our rRT-PCR method was able to detect down to 180 RNA copies/ml for all replicates with a N gene C_t_ values mean of 37.8 ± 1.2 (data not shown). Further concentrations under 180 copies/ml showed less detection with C_t_ values > 38. These data suggest that our optimized rRT-PCR method can detect down to 180 RNA copies/ml.

### Viral RNAs in UTM or RNase-free water is stable for a few days at 4 °C

**Fig. 4:**
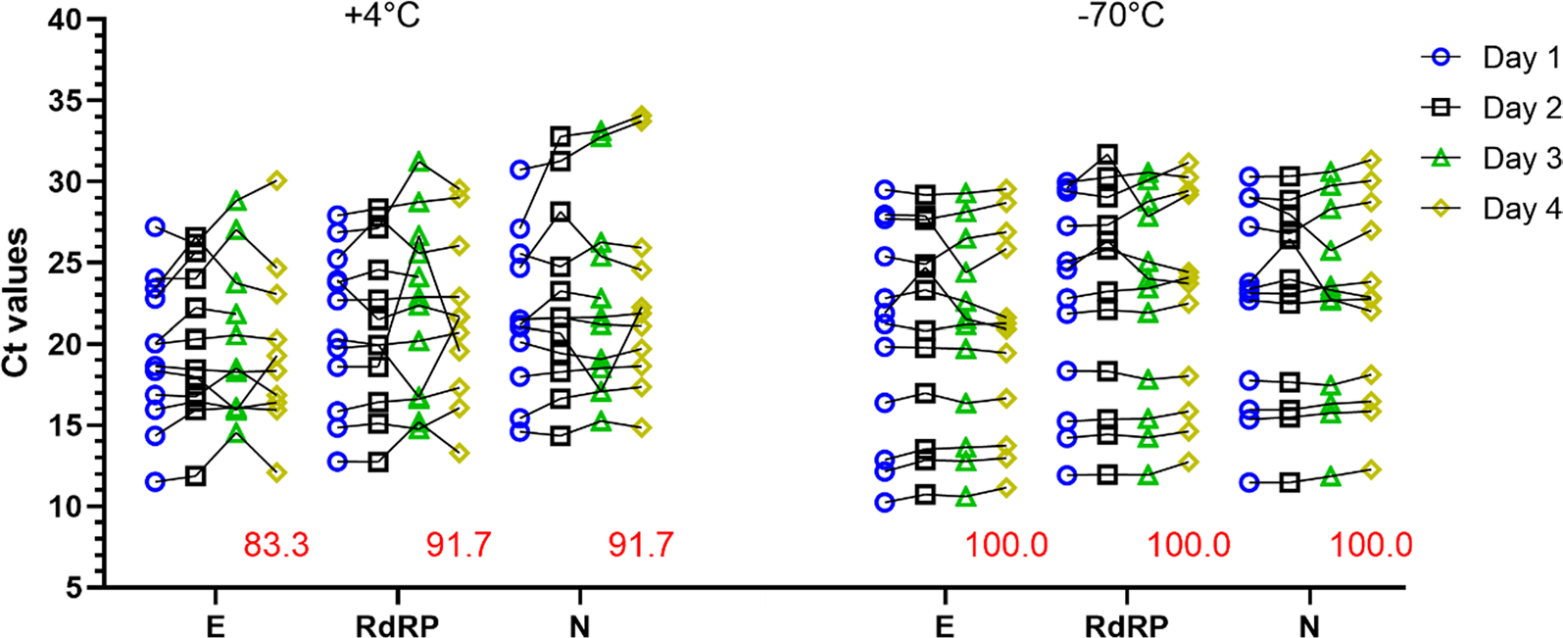
Effect of storage temperature on viral RNAs stability. E, RdRP and N genes from 4 non-extracted RNA samples in UTM (diluted 1/5), 4 non-extracted RNA samples in RNase-free water (dilution 1/2) and 4 RNA-extracted samples kept either at + 4 °C or − 70 °C were analyzed by rRT-PCR at day 1, day 2, day 3 and day 4. Red numbers indicate percentage of detection at day 4

Finally, since laboratories received thousands of samples a day, we sought to evaluate the stability of viral RNAs stored at 4 °C and − 70 °C. We performed rRT-PCR on 4 extracted-RNA samples, 4 non-extracted samples in UTM (diluted 1/5) and 4 in RNase-free water (dilution 1/2) at day 1, day 2, day 3 and day 4 (Fig. 4). Interestingly, freeze/thaw at − 70 °C cycle did not perturb viral RNAs detection since C_t_ values were steady at each time points and all specimens were detected as positive even on the fourth day. However, viral RNA kept at 4 °C showed some variations in detection and 1 UTM positive sample was missed on the fourth day (91.7% of detection). These data suggest that patient specimens should be frozen if they are not analyzed within 3 days.

## Discussion

In this study, we successfully optimized a direct rRT-PCR method to detect SARS-CoV-2 RNA in ONP swabs with the Allplex™ 2019-nCoV assay from Seegene Inc.

From sample sets 1 and 2, we evaluated the impacts of specimen dilution and thermal lysis on detection rates of direct rRT-PCR compared to manual RNA extraction as a standard reference. Detection was optimal using 1/5 diluted specimens collected in UTM and ½ diluted specimens collected in RNase-free water. While 1/5 dilution of specimens collected in UTM exhibited an efficiency of 93.3% of positive samples for three viral genes, two samples were missed. These two samples were detected after RNA extraction with C_t_ values greater than 30, suggesting that these samples had low viral loads. A thermal lysis treatment slightly improved the efficiency up to 96.6% detection by recovering one additional sample. The last remaining sample had high C_t_ values following RNA extraction (E: 34.4, RdRP: 37.3, N: 38.4), indicating that viral RNA levels were too low to be detected without RNA extraction. Although Merindol and colleagues showed that UTM is a suitable media for the amplification of SARS-CoV-2 without RNA extraction [8], we demonstrate that dilutions of samples are required to obtain reliable detection. UTM medium itself might be inhibitory for the rRT-PCR reaction, as suggested by the requirement of stronger dilutions for optimal detection on specimens stored in UTM compared to RNase-free water.

Thermal lysis improved detection rates and the number of detected genes in most conditions. This was especially notable in the case of 1/2 diluted UTM, where N gene detection rose from 93 to 97%, RdRP from 63 to 77% and E from 87 to 90%. Thermal lysis also improved detection rates of undiluted and 1/2 diluted RNase-free water from 90 to 100% and from 93 to 100%, respectively. Thermal lysis could contribute to specimen inhibitor denaturation, rather than a direct effect on viral template release, as supported by the loss of benefits at higher dilutions. Thus, optimized conditions for direct rRT-PCR include storage in RNase-free water as transport medium, 1/2 specimen dilution and thermal lysis treatment before rRT-PCR. In fact, these allowed detecting 30/30 positive specimens with minimal impact on C_t_ values as compared to manual RNA extraction. As a result of these findings, we switched from UTM as a transport medium to RNase-free water in our hospital.

Using sample sets 3, 4 and 5, our optimized direct rRT-PCR process was evaluated on a greater number of positive specimens with a wider range of C_t_ values. We also tested the impact of a PK pre-treatment on both detection rate, and invalid results rate using negative specimens.

In sample set 3, the detection rate increased marginally from 94.4% to 98.9% with addition of PK. In sample set 4, consisting exclusively of low to very low viral loads, the detection rate rose significantly from 53.3 to 76.7%. Finally, in sample set 5, 6.7% positive specimens were detected following PK addition. The increased sensitivity affected exclusively samples with C_t_ values > 33. Even in presence of PK, detection rates declined when C_t_ values were above 35, close to the detection limit of this test. Furthermore, PK treatment eliminated invalid results which represented 23.3% (28/120) results in sample sets 4 and 5 together. We propose that PK breaks down specimen-derived inhibitors, whose impact is greater when viral RNA levels are limited, affecting the IC amplification much more than the viral gene amplification.

In our experience, samples from newly infected patients often result in the detection of the three viral genes with C_t_ values lower than 25, suggesting a high viral titer. We have demonstrated that our protocol accurately detects specimens with low viral loads, detected at C_t_ values up to 36, and that detection rate declines beyond these levels. Interestingly, the majority of low viral load specimens are detected uniquely with the N gene using this assay. One possible explanation is a higher sensitivity of the N gene primers. All *coronaviruses* do nested transcription and generate couples of subgenomic mRNAs coding for different structural viral proteins. While the N gene sequence is present in all subgenomic mRNAs, E and RdRP are less represented [3]. We propose that specimens with only detected N gene of C_t_ values > 30 could be either non-infectious viral particles [9] or fragmented viral genomes that are no longer infectious. Finally, the majority of specimens with very low viral loads in this study are derived from patients in late stages of disease, or in the convalescent phase that were subject to Covid-19 detection for infection control purposes as mandated by the provincial health authorities in the province of Quebec. An abundant body of literature documents that infectivity from these patients is limited [10–15], and we conclude that missed positive samples using our method with C_t_ values above 35 have little clinical relevance.

## Conclusion

In conclusion, we successfully optimized the unextracted rRT-PCR process using the Seegene Allplex™ 2019-nCoV assay to detect SARS-CoV-2 RNAs in nasopharyngeal swabs. Our clinical laboratory currently tests for Covid-19 using ONP swabs collected in 2 ml of RNase free water which are diluted 1/2 with a solution of RNase free water and PK at a final concentration of 100 mg/ml. The samples are submitted to a 15 min 50 °C step to maximise PK activity and then to a thermal lysis step at 90 °C for 3 min which inactivates PK and viruses. Samples are then added to the PCR wells for amplification. We declare positive all specimens with N gene C_t_ values lower than 40, as established by the manufacturer’s instructions. The performance of this method is not only clinically acceptable but also confers cost savings and turnaround time advantages compared to automated extraction. We offer an alternative faster and cheaper rRT-PCR method with high efficiency of detection that could play an important role in the surveillance of Covid-19. In fact, Larremore and colleagues have suggested that effective surveillance depends on testing and the speed of reporting even more than high test sensitivity [16]. Our optimized rRT-PCR method reaches these criteria as we are able to perform analyses and report positive cases in few hours. Finally, we demonstrated that laboratories can safely store samples at 4 °C up to 3 days but should stock them at − 70 °C if they are unable to perform rRT-PCR within 3 days.

## Data Availability

Not applicable.

## Abbreviations

IC: Internal control
Ct values: Cycle threshold values
LoD: Limit of detection
ONP: Oro-nasopharyngeal
PK: Proteinase K
RdRP: RNA-dependent RNA polymerase
rRT-PCR: Real-time reverse transcription polymerase chain reaction
UTM: Universal transport media

## References

[1] Zhu N, Zhang D, Wang W, Li X, Yang B, Song J (2020). A novel coronavirus from patients with pneumonia in China, 2019. N Engl J Med.

[2] Corman VM, Landt O, Kaiser M, Molenkamp R, Meijer A, Chu DKW (2020). Detection of 2019 novel coronavirus (2019-nCoV) by real-time RT-PCR. Euro surveillance.

[3] Kim D, Lee JY, Yang JS, Kim JW, Kim VN, Chang H (2020). The architecture of SARS-CoV-2 transcriptome. Cell.

[4] Lalli MA, Langmade SJ, Chen X, Fronick CC, Sawyer CS, Burcea LC (2020). Rapid and extraction-free detection of SARS-CoV-2 from saliva by colorimetric reverse-transcription loop-mediated isothermal amplification. Clin Chem.

[5] Peng J, Lu Y, Song J, Vallance BA, Jacobson K, Yu HB (2020). Direct clinical evidence recommending the use of proteinase K or dithiothreitol to pretreat sputum for detection of SARS-CoV-2. Front Med.

[6] Mallmann L, Schallenberger K, Demolliner M, Antunes Eisen AK, Hermann BS, Heldt FH (2020). Pre-treatment of the clinical sample with proteinase K allows detection of SARS-CoV-2 in the absence of RNA extraction. bioRxiv.

[7] Seegene. AllplexTM 2019-nCoV Assay (Cat no. RP10250X / RP10252W) Instructions for Use. https://www.fda.gov/media/137178/download. 2020.

[8] Merindol N, Pepin G, Marchand C, Rheault M, Peterson C, Poirier A (2020). SARS-CoV-2 detection by direct rRT-PCR without RNA extraction. J Clin Virol.

[9] Klasse PJ (2015). Molecular determinants of the ratio of inert to infectious virus particles. Progress Mol Biol Transl Sci.

[10] Bullard J, Dust K, Funk D, Strong JE, Alexander D, Garnett L (2020). Predicting infectious SARS-CoV-2 from diagnostic samples. Clin Infect Dis.

[11] La Scola B, Le Bideau M, Andreani J, Hoang VT, Grimaldier C, Colson P (2020). Viral RNA load as determined by cell culture as a management tool for discharge of SARS-CoV-2 patients from infectious disease wards. Eur J Clin Microbiol Infect Dis.

[12] Zheng S, Fan J, Yu F, Feng B, Lou B, Zou Q (2020). Viral load dynamics and disease severity in patients infected with SARS-CoV-2 in Zhejiang province, China, January–March 2020: retrospective cohort study. BMJ.

[13] Huang CG, Lee KM, Hsiao MJ, Yang SL, Huang PN, Gong YN (2020). Culture-based virus isolation to evaluate potential infectivity of clinical specimens tested for COVID-19. J Clin Microbiol.

[14] Folgueira MD, Luczkowiak J, Lasala F, Perez-Rivilla A, Delgado R (2020). Persistent SARS-CoV-2 replication in severe COVID-19. medRxiv.

[15] Zou L, Ruan F, Huang M, Liang L, Huang H, Hong Z (2020). SARS-CoV-2 viral load in upper respiratory specimens of infected patients. N Engl J Med.

[16] Larremore DB, Wilder B, Lester E, Shehata S, Burke JM, Hay JA (2020). Test sensitivity is secondary to frequency and turnaround time for COVID-19 surveillance. medRxiv.

